# CRANAD-28: A Robust Fluorescent Compound for Visualization of Amyloid Beta Plaques

**DOI:** 10.3390/molecules25040863

**Published:** 2020-02-16

**Authors:** Kathleen Ran, Jing Yang, Anil V. Nair, Biyue Zhu, Chongzhao Ran

**Affiliations:** 1 Athinoula A. Martinos Center for Biomedical Imaging, Department of Radiology, Massachusetts General Hospital and Harvard Medical School, Room 2301, Building 149, Charlestown, Boston, MA 02129, USA; kathleenran@college.harvard.edu (K.R.); jyang37@mgh.harvard.edu (J.Y.); bzhu8@mgh.harvard.edu (B.Z.); 2 Center for systems biology, Program in Membrane, Biology, Division of Nephrology, Massachusetts General Hospital and Harvard Medical School, Boston, MA 02114, USA; AVNAIR@mgh.harvard.edu; 3 West China School of Pharmacy, Sichuan University, Chengdu 610041, China

**Keywords:** amyloid beta, plaque staining, fluorescent probe

## Abstract

CRANAD-28, a difluoroboron curcumin analogue, has been demonstrated in earlier reports to successfully label amyloid beta (Aβ) plaques for imaging both ex vivo and in vivo. CRANAD-28’s imaging brightness, ability to penetrate the blood brain barrier, and low toxicity make the compound a potentially potent imaging tool in Alzheimer’s research. In this study, the Aβ-labeling ability of CRANAD-28 was investigated in further detail using histological staining to assess different criteria, including stained Aβ plaque brightness, Aβ plaque size, and Aβ plaque number count. The results of this study demonstrated CRANAD-28 to be superior across all criteria assessed. Furthermore, CRANAD-28 and IBA-1 antibody were used to label Aβ-plaques and microglia respectively. Statistical analysis with Spearman regression revealed a statistically significant negative correlation between the size of labeled Aβ plaques and surrounding microglia density. This finding provides interesting insight into Aβ plaque and microglia dynamism in AD pathology and corroborates the findings of previous studies. In addition, we found that CRANAD-28 provided distinct spectral signatures for Aβs in the core and periphery of the plaques. Based on the study’s results, CRANAD-28 could be considered as an alternative standard for imaging Aβ-plaques in future research studies.

## 1. Introduction

The current, prevailing understanding of Alzheimer’s disease (AD) pathology is rooted in the amyloid hypothesis and Tau hypothesis. The amyloid hypothesis attributes cognitive decline and neurodegeneration to the accumulation and deposition of Aβ peptides within the brain [1,2,3]. According to the amyloid hypothesis, Aβ peptide is the causative agent which instigates neurofibrillary tangle formation, neuron death, and vascular damage, culminating in the disease’s most visible symptom, dementia [1,4,5]. Deposits of Aβ peptides form Aβ plaques, which are the characteristic hallmarks of AD. However, the role of Aβ plaques in AD pathology has increasingly come under attack as Aβ-clearing drugs have failed to halt or reverse cognitive decline in clinical trials [2,3]. Furthermore, the specific mechanism of neurodegeneration in AD remains unclear and is an area of continuing dispute among scientists [6,7,8]. Regardless, imaging Aβ plaques remains a crucial aspect of such studies and finding effective imaging tools can help clarify the pathological role of Aβ plaques in AD. 

CRANAD-28 is a small molecular compound derivatized from a curcumin scaffold. The compound is brightly fluorescent (quantum yield > 0.32 in PBS), with an excitation/emission peak of 498/578 nm in PBS solution [9]. CRANAD-28 has been previously shown to bind to monomeric, dimeric, and oligomeric Aβ peptides in vitro as well as bind to Aβ plaques in ex vivo histological staining. Furthermore, CRANAD-28’s ability to penetrate the blood brain barrier and low toxicity make the compound suitable for imaging Aβ plaques in vivo using two photon microscopy [9]. The capacity of CRANAD-28 to image Aβ plaques both in vivo and ex vivo makes the compound advantageous for streamlining experimental methods. In this study, we investigated the staining quality of Aβ plaque achieved by CRANAD-28 compared to that achieved by Thioflavin S, the currently gold standard for Aβ plaque staining [10,11,12].

## 2. Results

### 2.1. Brightness Comparison between CRANAD-28 and Thioflavin S

**Figure 1:**
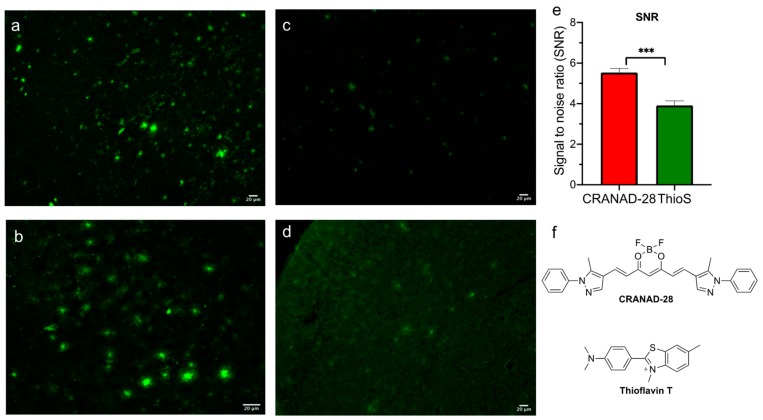
Representative images from APP/PS1 brain slice staining for brightness assay and SNR analysis. (**a**) A brain section stained with 20 uM CRANAD-28 (with a 20× object lens); (**b**) a brain section stained with 20 uM CRANAD-28 (with a 10× object lens); (**c**) a brain section stained with 20 uM Thioflavin S (with a 20× object lens); (**d**) a brain section stained with 1mg/mL Thioflavin S (with a 10× object lens); All images taken with fluorescence microscope and SPOT Advanced Imaging Software. Blue excitation filter with gain 4 and exposure time 335 ms. Scale bar: 20 μm; (**e**) quantitative SNR analysis results of staining with CRANAD-28 and Thioflavin S; (**f**) chemical structures of CRANAD-28 and Thioflavin T.

To evaluate the quality and robustness of CRANAD-28 Aβ plaque staining, Thioflavin S was used as the gold standard. The staining capability of CRANAD-28 was compared against Thioflavin S across several criteria. The first criterion was the brightness of labeled Aβ plaques. For the brightness assay, APP/PS1 transgenic mice brain tissue sections were incubated in matched concentrations of CRANAD-28 and Thioflavin S. Aβ plaques stained with CRANAD-28 were noticeably brighter than those stained with Thioflavin S at matched concentrations (Figure 1a–d). Brightness of the stained plaques was qualitatively assessed due to obvious differences upon visual inspection. Quantitative analysis was also used to calculate the signal to noise ratio (SNR) of both compounds. SNR_(CRANAD-28)_ = 5.54, while SNR_(Thioflavin S)_ = 4.27. The difference between the two SNRs calculated was determined to be statistically significant (*p* < 0.001) using a 2-sample equal variance t-test. These results matched our expectation that CRANAD-28 could provide brighter staining with a higher SNR.

### 2.2. Aβ Plaque Morphology and Quantification Assay

The next criterion assessed was the diameter of the stained Aβ plaques. Since CRANAD-28 has an excitation/emission spectrum of 498/573 nm and Thioflavin S 480/525 nm, tissue stained with either compound must be imaged using the blue excitation filter on the fluorescence microscope. Consequently, co-staining of Aβ plaques with both compounds could not be used as a means of assessment due to inability to distinguish the two compounds when using the same ex/em filters. Thus, antibody staining with 3D6 [13,14,15], a well-validated labeler of Aβ plaques, was used as a control against which CRANAD-28 and Thioflavin S staining were each compared. For this part of the study, the concentration of Thioflavin S was increased to 100 μM. APP/PS1 brain sections were first stained with the either Thioflavin S or CRANAD-28 followed by incubation with 3D6 antibody. The sections were then imaged under a fluorescence microscope using the green filter to detect CRANAD-28 or Thioflavin S and the red filter to detect 3D6. Plaque diameters were measured using ImageJ software and the ratios of ThioS:3D6 and CRANAD-28:3D6 were compared. 

Plaques labeled with 3D6 antibody were the largest and used as a control against which Thioflavin S and CRANAD-28 labeled plaques were compared. The diameters of Aβ plaques stained with CRANAD-28 demonstrated stronger correlation with those of Aβ plaques stained with 3D6 (R^2^ = 0.90 vs. 0.69). The average size of CRANAD-28, ThioS, and 3D6 labeling was 18-, 14-, and 20-micron respectively. The exact reason for the diameter difference between plaques stained with CRANAD-28 and Thioflavin S remains unclear. A possible explanation is that CRANAD-28 is able to bind to smaller Aβ species which make up the Aβ plaque periphery. This possibility is supported by the ability of CRANAD-28 to bind to soluble Aβ species in vitro.

**Figure 2:**
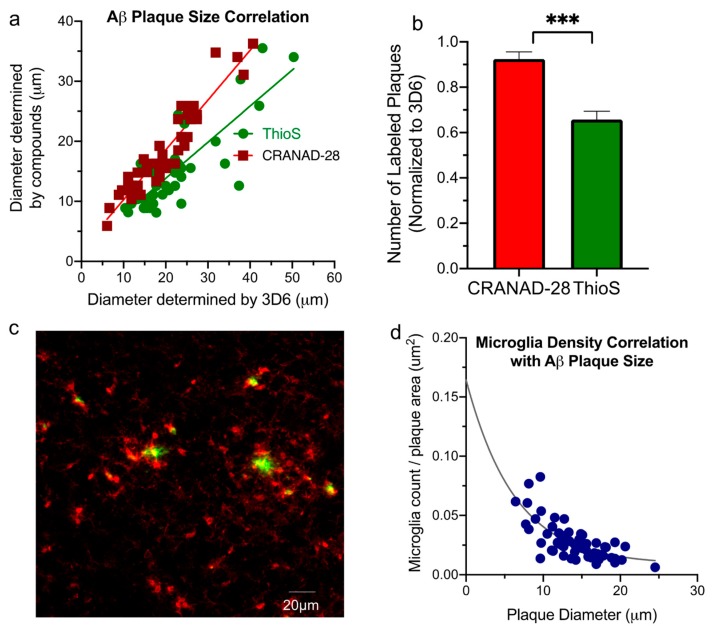
(**a**) Correlations between CRANAD-28 labeling, ThioS and 3D6 antibody staining. It can be seen that in the term of plaque size CRANAD-28 has a better correlation with 3D6; (**b**) consistency count comparation between CRANAD-28 and ThioS. Ratio of stained number of plaques compared as ratios CRANAD-28:3D6 and ThioS:3D6; *p* value *** < 0.001. (**c**) representative image of plaque staining with CRANAD-28 (green) and microglia staining with IBA-1(red); (**d**) correlation between diameter of plaques labeled with CRANAD-28 and microglia density taken as microglia count divided by plaque area. Rs = −0.70, *p* < 0.001.

Using the same co-staining method previously described, the number of plaques successfully labeled by CRANAD-28 and Thioflavin S was also compared. Images of Aβ plaques in the cortex were selected at random. The number of 3D6 antibody stained plaques was used as a control, since antibody staining was found in this study to be most inclusive in number of plaques stained. From the acquired images, the number of plaques stained by either compound was calculated as a ratio over the number of plaques stained by 3D6. Plaques were manually counted. On average, CRANAD-28 successfully labeled more plaques than Thioflavin S (Figure 2b).

### 2.3. Aβ Plaque and Microglia Staining

Recent evidence indicates that Aβ plaques transform in a dynamic way. As they develop, the plaques have a different impact on the environment in which they reside. Data shows that at the early stage of growth, Aβ plaques lead to higher toxicity than in the later/mature stage. Based on recent experimental findings, we hypothesize that plaques of high toxicity are “active” plaques, while plaques of low (or no) toxicity are “silent” plaques [16], and the abundance of “active plaques” may have better correlations with the severity of AD than the number of total plaques. Considering the excellent staining capacity of CRANAD-28, we further investigated the relationship between plaque size and the density of surrounding microglia.

To investigate the relationship between microglia density and plaque size, APP/PS1 brain sections were first incubated in CRANAD-28 followed by staining with IBA-1 antibody, the widely accepted biomarker for microglia. The blue filter was used to detect Aβ plaques stained by CRANAD-28, and the green filter was used to detect microglia stained with IBA1 antibody. Microglia density was calculated by taking the total number of microglia surrounding an individual plaque over the area of the plaque. ImageJ software was used to stack images as well as keep track of counted microglia using ROI manager. For the microglia count, the region of interest was defined as the circular area 2× the diameter of the plaque. As we expected, the smaller plaques generally have higher microglia density around them (Figure 2c). A Spearman regression correlation coefficient of −0.70 (*p* < 0.001) was calculated for Aβ plaque area versus surrounding microglia density. 

### 2.4. Spectral Signatures of CRANAD-28 for Core and Periphery of the Aβ Plaques

**Figure 3:**
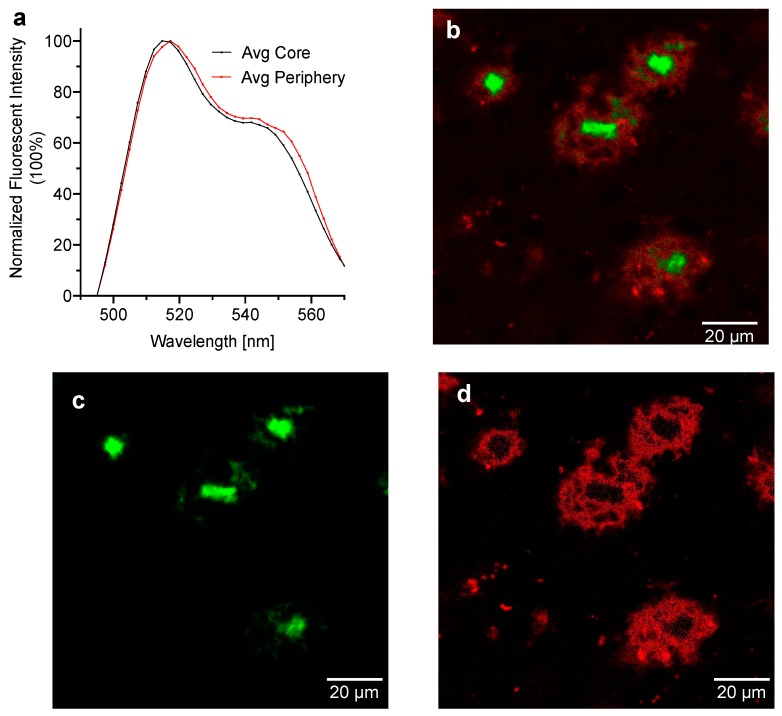
(**a**) Emission spectra peaks from 5× FAD transgenic mouse brain sections stained with CRANAD-28; (**b**) Merged confocal microscope images Aβ plaques from the unmixed image of core (green) and the unmixed image of periphery (red). 488 nm excitation, 40× objective, zoom 2×; (**c**) confocal microscope image of the unmixed core component using the extracted spectra; and (**d**) confocal microscope image of the unmixed periphery component using the extracted spectra.

For spectrum analysis of Aβ plaques stained with CRANAD-28, images were taken using the 40× oil immersion lens of a Nikon A1 confocal microscope. For each imaged plaque, the spectrum emission from 4 regions of interest in the core and 4–8 regions of interest in the periphery were measured using the Spectrum Profile function in NIS Elements. The extracted spectra from the core and periphery were averaged and stored. We found that there were two distinguishable signatures from the spectra. First, the averaged emission from the core had a maximum peak in intensity at 512 nm, while the averaged emission spectrum from the periphery had a maximum peak in intensity at 520 nm (Figure 3a). Another obvious signature could be seen from 540–570 nm, and the relative intensity from the peripheral area was higher than that from the core. Spectral un-mixing was used to assign the different peaks present in the signatures to either the core or peripheral regions of the plaques. For spectral un-mixing, the stored spectra previously extracted from the core and peripheral regions served as reference spectra. A visible difference in emission peak wavelength was observed between the core and periphery regions of the plaques, indicating that CRANAD-28 exhibits differential binding to Aβ species within the plaques (Figure 3b,c). Our data also suggested that CRANAD-28 could be used to investigate the pathological effects of the Aβ species from the core and periphery. While the spectra from the core and periphery are not the same spectra from the solution testing, signature differences could be identified in the range of 530–570 nm, which responds to the fluorescence of CRANAD-28. In this experiment, we used the spectral unmixing function from the software for the microscope. We believe that better algorithms will be able to tease out larger signature differences.

## 3. Discussion

In the broader scope of biological mechanisms driving AD pathology, our microglia correlation results support the idea that Aβ plaques are dynamic rather than static structures. A number of recent studies have demonstrated increased interest in Aβ plaques as potentially dynamic sites of AD pathology [17,18]. Furthermore, numerous studies suggest that Aβ plaques grow and expand dynamically throughout AD progression. This could potentially explain several postmortem clinicopathological studies reporting poor correlation between Aβ plaque loading and severity of sporadic AD [19,20,21,22]. Consistent with the previous studies, the significant inverse relationship between Aβ plaque size and microglia density in this study suggests that perhaps smaller plaques are more active sites of pathology, inducing more neuronal degeneration than larger plaques [23,24]. This finding indicates that the evolution of Aβ plaque function during the plaque growth process may be an interesting avenue for future research. 

Based on the finding that staining with CRANAD-28 generally results in larger amyloid beta plaques, we speculate that CRANAD-28 exhibits binding to soluble Aβ species such as oligomers and monomers. Although the structures of monomeric and oligomeric Aβ species require electron microscopy for visualization, it is possible that upon labeling with CRANAD-28 they can cause a measurable increase in the diameter of imaged plaques. Aβ oligomers are of particular interest in Alzheimer’s disease pathology. The Aβ oligomer toxicity hypothesis postulates that Aβ oligomers, as opposed to the plaques, instigate neuron damage in Alzheimer’s disease. Previous studies have demonstrated that Aβ oligomers instigate synapse loss, inflammation, and oxidative damage in neuron cells [6,25]. Our speculation that CRANAD-28 possesses an ability to bind to soluble species is supported by our spectral unmixing results, in which the emission spectrum peak from the periphery region of the plaque occurs at a slightly longer wavelength than the emission spectrum peak from the core region. A possible cause for the observed difference is the difference in hydrophobicity of the oligomers versus aggregates. Since aggregates are more hydrophobic, the emission spectrum from the aggregate-dense core would be expected to have a greater blue shift upon binding with CRANAD-28. 

The excellent staining capabilities of CRANAD-28 determined in this study indicate that the compound is a superior candidate for Aβ plaque labeling and widely applicable for different studies of AD pathology. The compound’s comprehensive staining of Aβ plaques makes it a potent tool for studying Aβ plaque development over the progression of Alzheimer’s disease, allowing for more detailed and informative data collection. Furthermore, unlike Thioflavin S and 3D6 antibody, CRANAD-28 can be used for in vivo imaging, making it possible to track Aβ plaque development in living mouse models. Based on our studies, we can conclude that CRANAD-28 is a robust fluorescent compound for the visualization of Aβ plaques.

## 4. Materials and Methods

All the chemicals employed in the syntheses were purchased from commercial vendors and used without further purification. The pH of the PBS buffer was 7.4. CRANAD-28 was prepared according to the previously reported procedure [9]. Transgenic female APP/PS1 mice and age matched wild-type female mice were purchased from Jackson Laboratory. A brain of a 24-month old APP/PS1 mouse was sacrificed and the brain was extracted. All animal experiments were approved by the Institutional Animal Use and Care Committee at Massachusetts General Hospital.

### 4.1. Transgenic Mouse Brain Slide Preparation

A 24-month old APP/PS1 mouse was sacrificed, and the brain was extracted. The brain was fixed with 4% formalin for 48 hours, and washed with PBS buffer 3–4 times. The brain was then placed in 30% sucrose PBS buffer and soaked at 4 °C until it sunk to the bottom of the container. The sunken brain was removed from solution and imbedded in OCT via gradual freezing over liquid nitrogen. The OCT brain block was cut into 20-μm thick slices with a cryostat machine.

### 4.2. Slide Staining and Fluorescence Imaging with CRANAD-28 and Thioflavin S

Brain sections were mounted onto a glass slide and fixed in 4% formalin for 5 min and washed with PBS buffer twice. A hydrophobic barrier pen was used to draw a waterproof barrier around the mounted sections. Sections were incubated in a solution of CRANAD-28 (20 uM, 50% ethanol) followed by washing with distilled water 3–4 times. After drying at RT, the slide was ready for fluorescence microscope imaging. For thioflavin S staining, the same staining method was used with a thioflavin S solution 20 μM. Images were captured by using a microscope (Nikon Y-FL model E400, Nikon Instruments Inc, Melville, NY, USA) with advanced SPOT imaging software. The fluorescence intensity and size of Aβ plaques and microglia cells were quantified by using ImageJ software (National Institute of Health, USA).

### 4.3. Slide Staining with 3D6 Antibody and IBA-1 Antibody

Brain sections were mounted onto glass slides, fixed in 4% formalin for 5 min, washed twice for 5 min in 50% ethanol, and washed twice for 5 min in distilled water. Then, sections were blocked with 20% Goat serum for 1 hour at room temperature. After, sections were incubated with anti-Aβ antibody 3D6 (diluted in a diluting buffer: TBS containing 0.4% Triton X-100, 1% goat serum and 2% BSA) overnight at 4 °C. After 3 × 10 min washes with diluting buffer, sections were incubated with secondary AlexaFluor-555 goat anti-rabbit antibody for 4 h at RT. Sections were washed 3 × 10 min in TBS. Fluorescent Vectashield mounting medium was applied to the sections, and sections were covered with a coverslip and nail polish airtight seal. For IBA-1 antibody staining, the same protocol was followed using IBA-1 as the primary antibody. The primary antibody concentration used was 1:500 and the secondary antibody concentration used was 1:1000. Slides were stored at 4 °C in a dark area until observation and analysis. Florescence images were observed using the Nikon Y-FL model E400 microscope. 

### 4.4. Confocal Microscope and Spectrum Extraction

Stained brain sections were imaged using a Nikon A1 confocal microscope (Nikon Instruments Inc, Melville, NY, USA). All images were taken using the 40× oil objective lens and 457 nm excitation. Extracted spectra from ROIs within the core and periphery were averaged and stored using the Spectrum Profile function. Spectral unmixing was also performed with the Spectrum Profile function.
